# Scaling-Up Techniques for the Nanofabrication of Cell Culture Substrates via Two-Photon Polymerization for Industrial-Scale Expansion of Stem Cells

**DOI:** 10.3390/ma10010066

**Published:** 2017-01-13

**Authors:** Davide Ricci, Michele M. Nava, Tommaso Zandrini, Giulio Cerullo, Manuela T. Raimondi, Roberto Osellame

**Affiliations:** 1 Department of Chemistry, Materials and Chemical Engineering “Giulio Natta”, Politecnico di Milano, 20133 Milano, Italy; davide2.ricci@mail.polimi.it (D.R.); manuela.raimondi@polimi.it (M.T.R.); 2 Istituto di Fotonica e Nanotecnologie (IFN)-CNR and Department of Physics, Politecnico di Milano, 20133 Milano, Italy; tommaso.zandrini@polimi.it (T.Z.); giulio.cerullo@polimi.it (G.C.); roberto.osellame@polimi.it (R.O.)

**Keywords:** two-photon laser polymerization, microfabrication, synthetic nichoids, stem cell expansion, pluripotency maintenance, biomimetics

## Abstract

Stem-cell-based therapies require a high number (10^6^–10^9^) of cells, therefore in vitro expansion is needed because of the initially low amount of stem cells obtainable from human tissues. Standard protocols for stem cell expansion are currently based on chemically-defined culture media and animal-derived feeder-cell layers, which expose cells to additives and to xenogeneic compounds, resulting in potential issues when used in clinics. The two-photon laser polymerization technique enables three-dimensional micro-structures to be fabricated, which we named synthetic nichoids. Here we review our activity on the technological improvements in manufacturing biomimetic synthetic nichoids and, in particular on the optimization of the laser-material interaction to increase the patterned area and the percentage of cell culture surface covered by such synthetic nichoids, from a low initial value of 10% up to 88% with an optimized micromachining time. These results establish two-photon laser polymerization as a promising tool to fabricate substrates for stem cell expansion, without any chemical supplement and in feeder-free conditions for potential therapeutic uses.

## 1. Introduction

### 1.1. Rationale Underlying Industrial-Scale Expansion of Stem Cells and Limitation of Feeder-Cell Layers and Exogenous Conditioning

Stem cell-based therapies represent the most challenging and, potentially, the most successful applications for stem cells (SCs) [1]. Multipotent adult stem cells, including mesenchymal stem cells (MSCs) and adipose stem cells, are likely to be important sources for such therapies both because of the ease of access and the autologous derivation, which involves a low risk of infection and low immunoresponse from the host [2,3]. In addition, induced pluripotent stem cells (iPSCs), which can be obtained from the genetic reprogramming of somatic cells to their pluripotent stage and then induced towards a specific cell phenotype [2,4], represent a further promising source because they allow ethical issues related to embryonic stem cells (ESCs) to be overcome. However, several limitations need to be overcome prior to the successful exploitation of SC-based therapies in clinics. For example, the low efficiency of iPSC reprogramming [5] and the average number of cells needed for cell therapies and regenerative strategies is typically in the order of 10^6^ to 10^9^ cells, whereas the number of SCs from 4 mL-bone-marrow aspirate is around 700 cells [6]. Therefore, a (minimal) in vitro cell manipulation, including expansion in order to achieve high cell densities by ensuring either multi/pluripotency maintenance or differentiation towards the correct lineage to meet clinical demands is necessary.

In this context, efforts have focused on optimized culture media with a well-defined composition. Such media include additives and small molecules that inhibit signaling pathways associated with cell death and differentiation. For example, Valamehr and colleagues [7] reported the maintenance of a homogeneous population of undifferentiated human iPSCs by supplementing the standard culture media with “SMC4” containing the cell pathway inhibitors Glycogen Synthase Kinase 3, MAPK/ERK Kinase, Rho-Associated Protein Kinase, and Transcription Growth Factor-beta. The same research group derived a culture medium, namely a fate maintenance medium, containing SMC4 and leukemia inhibitory factor (LIF), and basic Fibroblast Growth Factor (bFGF) [8]. They reported that (mouse) ESCs had a lower expression of genes related to three germ layers differentiated with respect to those cells expanded on conventional feeder layers. Another example relevant to the development of protocols of chemically-defined media for the maintenance and expansion of (mouse) ESCs consisted in growing cells in suspension as spheroids in LIF-bFGF-conditioned medium to ensure long-term pluripotency and very few differentiated cells [9].

Examples of commercially available chemically-defined media for a feeder-free (FF) culture of human iPSCs are PluriSTEM (Merck Millipore, Darmstadt, Germany), StemPro (Invitrogen, Carlsbad, CA, USA), mTeSR1 (STEMCELL Technologies, Vancouver, BC, Canada), Pluripro (Cell Guidance Systems, St. Louis, MO, USA), Stemline (Sigma, St. Louis, MO, USA), and Essential-8 (Invitrogen, Carlsbad, CA, USA) [10]. Despite all the advantages in terms of expansion efficiency, the long-term clinical side-effects on human beings in the case of SC transplantation needs to be assessed in advance. For example, LIF has been proven to increase cancer expansion and metastasis in human osteosarcoma and carcinoma, enhancing the phosphorylation of signal transducers and activators of transcription-3 [11], as well as mediating the proinvasive activation of stromal fibroblasts [12]. In addition, such culture media typically may contain animal-derived components, (e.g., fetal bovine serum and/or bovine serum albumin) to enhance SC growth. The serum composition as well as the presence of differentiation factors are generally undefined, so that the serum lots need to be screened and certified for SC use, resulting in high direct/indirect costs [9]. For these reasons, serum-free and additive-free culture conditions are preferable.

For human pluripotent stem cells, an animal feeder cell layer (e.g., mouse embryonic fibroblasts) is usually employed to support cell adhesion, survival, and self-renewal [3,13]. However, the biological products secreted by such xenogeneic feeder-cells are also a source of pathogens and mycoplasma contaminations which might induce an acute immune response in the host upon SC transplantation. To prevent such issues, human feeder cell layers have been introduced, such as recombinant E8 fragments of laminin isoforms [14], recombinant human laminin 511 [15,16], and clinical grade human foreskin fibroblasts [17,18]. Nevertheless, there are still several drawbacks related to the cost, the high lab-to-lab variability, high batch variability, as well as a limited scalability. An additional issue is the persistence of viral and non-viral infections from allogenic materials, the complexity in the maintenance of cells, the isolation and separation of a pure population of SCs with respect to feeder-cells. Finally, feeder-cells release a not-well defined variety of factors which may affect the interpretation of the biological results [3] and lead to safety issues when the SCs are transplanted. An example of FF culture consists in different extracellular matrix (ECM) extracts such as Matrigel^®^ [19] derived from mouse sarcoma cell basement membrane. Matrigel^®^ is currently the gold standard among ECM culture substrates for expanding SCs in feeder-free conditions. Nevertheless, exposure to animal-derived pathogens, xenogeneic components, and immunogenic epitopes would make these cells unsafe for clinical applications in humans [3]. Recently, a serum- and xeno-free substrate composed of conditioned medium from human dermal fibroblasts for long-term expansion of human ESCs and human iPSCs has been reported, named “RoGel” [20]. Despite decreasing the risk of animal-derived pathogen contamination, this system may have limitations, including high batch variability, costs, and the risks associated with human-derived pathogens and allogenic epitopes which severely limit potential therapeutic applications in humans.

Besides, the development of well-defined culture media, feeder-free and xeno-free substrates for cell expansion in vitro, researchers have focused on culture substrates that mimic at least one of the features of the physiological microenvironment surrounding cells. Increasing evidence has shown that, despite their extensive usage, conventional substrates such as culture polystyrene or glass dishes do not resemble the in vivo milieu. Thus, all the complex interactions that occur in vivo are impaired [21,22,23]. Various strategies have been developed to overcome these limitations and improve substrates for cell culture [13]. In order to recreate feeder free substrates for expansion and pluripotency maintenance of human stem cells, Park and colleagues developed a chemically-defined coating (i.e., polydopamine-mediated oligovitronectin) for conventional polystyrene culture plates, thereby promoting human ESC self-renewal and pluripotency maintenance [24]. Another approach is to mimic the physical and mechanical features of the natural microenvironment, including the nanotopography and the three-dimensional (3-D) geometry of such substrates [25,26,27]. A “smart” 3-D substrate, may be able to instruct cells towards the right fate, limiting and, whenever possible, avoiding any medium additive or supplement and/or xenogeneic-factor. There would be enormous benefits in terms of safety and risk mitigation during in vitro manipulation, thus leading to a potential industrial-scale expansion of cells for therapeutic purposes [10].

### 1.2. State of the Art in the Use of 3-D Substrates for the Expansion of Stem Cells

Several studies have proposed natural and synthetic polymers to recreate 3-D culture conditions, such as gelatin [28], collagen-I [29], fibronectin [30], Polyethylene-glycol (PEG) [31], Poli-L-Glycolic Acid [32,33], hyaluronic acid [34], vitronectin [35], poly(N-isopropylacrylamide)-PEG [36,37], and carboxymethyl-hexanoyl chitosan [38] in the form of hydrogels, nanofiber scaffolds and other 3-D structures [21,26]. These studies have shown that SCs could maintain a proliferative and undifferentiated state only by exploiting the biophysical cues offered by the surrounding environment. Besides established fabrication processes (e.g., solvent casting-particulate leaching, gas foaming, electrospinning, and fiber bonding), rapid prototyping methods (e.g., 3-D printing, fused deposition modelling, selective laser sintering, and electron beam lithography) have been increasingly used in scaffold fabrication because of the high resolution that can be achieved [39]. However these techniques, based on a layer-by-layer approach, require the use of (multiple) masks, and the complex 3-D architectures are difficult to manufacture [40]. A recent work proposed a fabrication method that produces poly(ε-caprolactone) (PCL) nanofibers over a large culture surface, by pressing the PCL substrate against a femtosecond laser fabricated glass mold to study the effects of the mechanics on cell fate [41].

### 1.3. State of the Art in the Use of 2PP for the Nanofabrication of Substrates for Cell Culture

An alternative scaffolding fabrication method is two-photon laser polymerization (2PP), a direct laser writing technique with three-dimensional (3-D) capabilities and a spatial resolution down to 100 nm [42,43,44]. 2PP employs a photosensitized resist, where simultaneous absorption of two photons from a near-infrared ultrashort laser pulse triggers a photochemical process which results in the cross-linking of monomers and oligomers. The two-photon absorption mechanism enables selective polymerization in a small volume around the focus, known as volume element or voxel. This enables sub-diffraction limited resolution in both the lateral and axial directions as well as 3-D fabrication capabilities at different depths by adjusting the laser focus with little or no collateral effects. Examples of photopolymers used in 2PP are organically modified ceramics, epoxides (e.g., SU-8), and acrylic monomers whose biocompatibility has been assessed [40,45]. Recent studies have shown that 3-D geometries can be manufactured by 2PP in PEG-diacrylate, poly lactic acid (PLA) and PLA-poly-ε-caprolactone copolymer [46,47,48]. In our previous work, we manufactured 3-D “structurally” biomimetic synthetic niches for MSC culture [49,50,51,52]. Evidence of spontaneous lineage commitment was observed in the monolayer culture surrounding the structural niches, but not inside the niches, thus suggesting that structural niches were able to direct SC homing, proliferation, and multipotency maintenance. However, the quantitative biological measurements were inevitably diluted because of the limited percentage (10%) of cell culture surface covered by the structural niches [52].

In this paper, we review our activity on the technological improvements in manufacturing the 3-D “structurally” biomimetic synthetic niche system for SC culture. In particular, more than on the biological results, we focus here on the optimization of the laser-material interaction that we performed in the last years to increase the patterned area and the percentage of cell culture surface covered by such 3-D synthetic niches, reaching the most recent result of 88% surface coverage with an optimized micromachining time. The aim of this study is to obtain a greater number of cells experiencing the 3-D microenvironment provided by the increased number of niches and, therefore to obtain quantitative, reliable, biological data to conclusively demonstrate the effect of the 3-D architecture on the SC fate.

## 2. Materials and Methods

### 2.1. Description of Photosensitive Material and Photo-Initiator

Synthetic niches were directly fabricated by 2PP in the commercially available SZ2080 photoresist, composed of a sol-gel-synthetized silicon (S)-zirconium (*Z*) hybrid inorganic-organic resin (hereafter called SZ2080) (Maria Farsari, IELS-FORTH, Heraklion, Greece) [53]. The main components of SZ2080 are methacryloxypropil trimethoxysilane and zirconium propoxide with the addition of 1% concentration of Irg photoiniziator (Irgacure 369, 2-Benzyl-2-dimethylamino-1-(4-morpholinophenyl)-butanone-1) [50]. SZ2080 has many advantages, such as biocompatibility [45,49], chemical and electrochemical inertia, long-term stability, good optical transmission, and mechanical stability after polymerization due to low shrinkage compared to other commercial photoresists [45].

### 2.2. The Laser Fabrication Set-Up

Synthetic niches were directly written onto circular glass cover slips with a 150-μm thickness and 12-mm diameter (Bio-Optica, Milan, Italy). Initially, we used a home-built Ti:Sapphire femtosecond laser (87 MHz repetition rate, 40 fs pulse duration, up to 400 mW average power) and a 3-D piezo positioning system (P-611.3 NanoCube, Physik Instrumente, Karlsruhe, Germany) with a travel range of 100 μm in each direction. Subsequently, we changed both the femtosecond laser and the positioning system. We used a home-built cavity-dumped Yb:KYW mode-locked system with a 1030 nm wavelength, generating pulses with 300-fs duration, energy up to 1 μJ and 1-MHz repetition rate, corresponding to 1 W of average power. The laser beam was focused on samples with a 1.4 numerical aperture 100× oil immersion objective (Plan-Apochromat, Carl Zeiss, Oberkochen, Germany). A power control stage, consisting of a polarizing beam splitter and a rotating waveplate, was used to tune the laser output power, while a mechanical shutter (LS Series, Uniblitz Electronics, Rochester, NY, USA), was used to switch the laser beam impinging on the sample on and off. A computer-controlled three-axis brushless motion stage (ANT130, Aerotech, Hanover, MD, USA) was used to control the position of the sample with respect to the laser focus in the two plane dimensions (*X* and *Y*), and to move the vertical position of the laser focus (*Z*) with respect to the sample plane, via specific motion controller software (Automation 3200 CNC Operation Interface, Aerotech, Hanover, MD, USA). This fabrication system has a very high precision and resolution down to ≈5 nm, maximum displacement in the order of tens of cm, and a very high speed up to 150 mm∙s^−1^. Its characteristics are thus ideal for patterning large areas through 2PP. A two-axis stage moves the sample in the *X*-*Y* plane, while a third axis, moving the focusing objective, scans along the *Z* direction. The system controller enables the three axes to move simultaneously, thus enabling 3-D writing at a constant speed. A gimbal mechanical system (Gimbal Mounts GM100/M, Thorlabs Inc., Newton, NJ, USA) fixed to the *X*-*Y* motion stages, was used to tilt the sample and to set it perpendicular with respect to the laser beam. A CMOS camera (DCC1545M, Thorlabs Scientific Imaging (TSI), Austin, TX, USA) and a beam splitter were used to visualize on computer software (uEye Cockpit, 4.71, IDS Imaging Development Systems GmbH, Obersulm, Germany) the image of the working field, back-lit by a red-light LED. The on-line vision of the polymerized lines enabled the structure fabrication to be accurately positioned in *X*-*Y* and *Z*. Prior to laser exposure, the SZ2080 photoresist was manually placed on glass coverslips baked at 105 °C (ramp starting from room temperature) for 1 h. Samples with the photoresist were mounted on a parallelepiped metallic custom-made holder and exposed to the laser in order to identify the microstructures by 2PP. To remove the unpolymerized regions, the samples were immersed in a 50% (*v*/*v*) 3-pentanone, 50% (*v*/*v*) isopropyl alcohol solution (Sigma-Aldrich, St. Louis, MO, USA). The fabricated 3-D niche substrates were then imaged first by optical microscopy (Eclipse ME600, Nikon, Tokyo, Japan), and then by scanning electron microscopy (SEM, Phenom Pro, Phenom World, Eindhoven, The Netherlands). All SEM observations were carried out at 5 kV.

### 2.3. Description of the Up-Scaling Techniques

**Figure 1:**
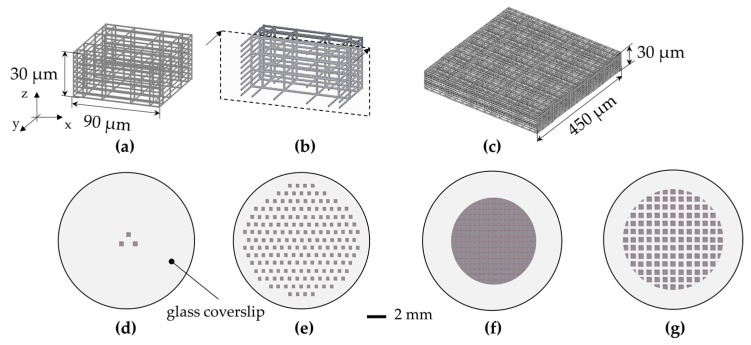
The nichoid culture substrate. (**a**) CAD of the nichoid elementary unit; (**b**) Cross-section of the nichoid; (**c**) CAD of the matrix of nichoids, consisting of 5 × 5 elementary nichoids. Scale-up approach: (**d**) the culture substrate composed of three elementary nichoids; (**e**) The substrate composed of approximately 367 elementary nichoids; (**f**) The culture substrate covered with continuously-packed nichoids (CPN), resulting in 3500 adjacent elementary nichoids; (**g**) The culture substrate covered with a fractionated supermatrix of nichoids (FSN) including 218 matrixes at a distance of 30 µm.

In order to obtain quantitative and reliable biological data to assess the effect of the 3-D architecture on the stem cell fate, we increased the surface of the culture substrate covered by the 3-D synthetic niches to obtain a greater number of cells experiencing the 3-D microenvironment provided by the niches. We thus adopted the following strategy. Firstly, we initially optimized the fabrication process of the elementary 3-D niches, which we named ‘nichoids’. Each nichoid was 30 μm high and 90 × 90 μm in transverse dimensions, and consisted of a lattice of interconnected lines, with a graded spacing between 10 and 30 μm transversely and a uniform spacing of 15 μm vertically. Each nichoid was surrounded by four outer confinement walls formed by horizontal lines spaced by 5 μm, resulting in gaps of 1 μm (Figure 1a,b). Secondly, the nichoids were arranged at the vertexes of an equilateral triangle with 200 µm sides (Figure 1d) [49]. Then, to increase the substrate coverage to the whole slide surface, the nichoids were arranged at the vertexes and at the center of a hexagon with a 300 μm side (Figure 1e) and the total number of elementary nichoids was increased to 367 μm [49,52]. However, the increased number of nichoids led to an increased manufacturing time, thus the manufacturing parameters (laser power and scan speed) needed to be optimized (see Section 3.1). This last configuration however covered only a 10% fraction of the cell culture surface with nichoids, resulting in a large percentage of SCs experiencing the 2-D environment of the glass slide surface. Thus, to further increase the coverage, we designed a very large scaffold that covered a circular area with a 3 mm radius, composed of continuously-packed nichoids (CPN) with about 3500 elements, where each nichoid shared its external walls with the adjacent ones (Figure 1f).

Despite the large surface covered with this approach, there were several drawbacks, including the low structural stability (see Section 3.2). To overcome these issues, we fractionated the large scaffolds into submatrixes with 5 × 5 nichoids (Figure 1c) with a small spacing of 30 µm between adjacent matrixes, resulting in a fractionated supermatrix of nichoids (FSN) including 218 matrixes (Figure 1g). In this last configuration, the entire structure was reinforced by an external 4-µm high base wall, which anchored the structure to the underlying glass surface and forced the cells to enter the nichoids from the top. In addition, to ensure the robustness of the structures, the pillars were reinforced by a double scan irradiation with a lateral shift of 0.5 µm. To prevent mechanical damage to the shutter, due to frequent opening and closing, our control software quickly relocated the laser focus inside the glass substrate to avoid photopolymerization when positioning the writing beam in the *X*-*Y* plane.

## 3. Results and Discussion

### 3.1. Parameter Optimization of 2PP-Engineered Elementary Nichoids

Up-scaling the synthetic nichoid culture system to obtain more nichoid-cultured SCs that could be compatible with clinical demands had various limitations, including the long machining time required to fabricate an elementary nichoid. Initially, we were only able to manufacture a few (e.g., three) elementary nichoids for each culture substrate. Using the Ti:Sapphire laser and the piezo positioning system, we were limited to a processing speed of 10 µm·s^−1^, thus requiring a long manufacturing time of 30 min per nichoid (Table 1, column A).

To speed up the fabrication, we used a different femtosecond laser and translation stages (see Section 2.2). In this new configuration we were able to work in new processing conditions with writing speeds increased by two orders of magnitude, thus fabricating an elementary nichoid in about 30 s (Table 1, column B). In these new processing windows, we optimized the laser manufacturing parameters (laser power and scan speed) to identify the processing windows that would provide stable structures (Table 2). We tested several combinations of laser power and scan speed ranging from 12–15 mW and 1–10 mm∙s^−1^, respectively.

**Figure 2:**
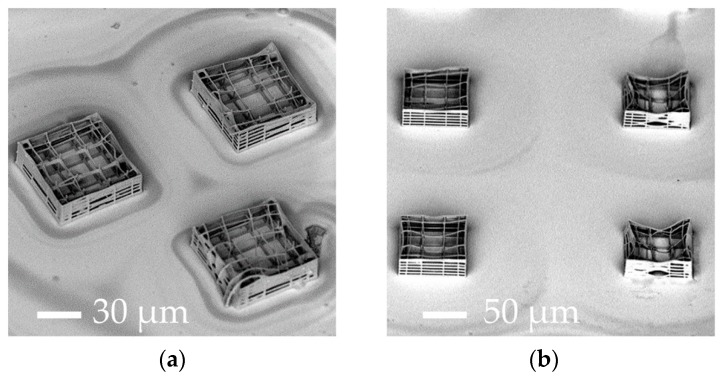

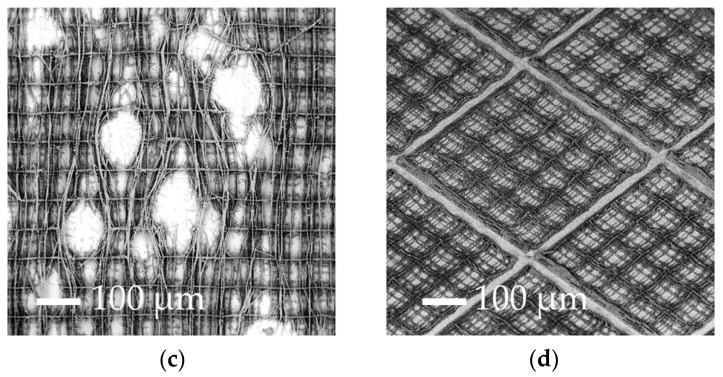
SEM images of the micro-fabrication before the optimization. Instability and deformation on (**a**) three elementary nichoids; (**b**) on the substrate composed of 367 elementary nichoids; (**c**) Collapse of the culture substrate covered with continuously-packed nichoids (CPN), more likely due to cavitation phenomena and vibration during the manufacturing process; (**d**) Instability issues in the culture substrate covered with a fractionated supermatrix of nichoids (FSN), including 218 matrixes at a distance of 30 µm.

**Figure 3:**
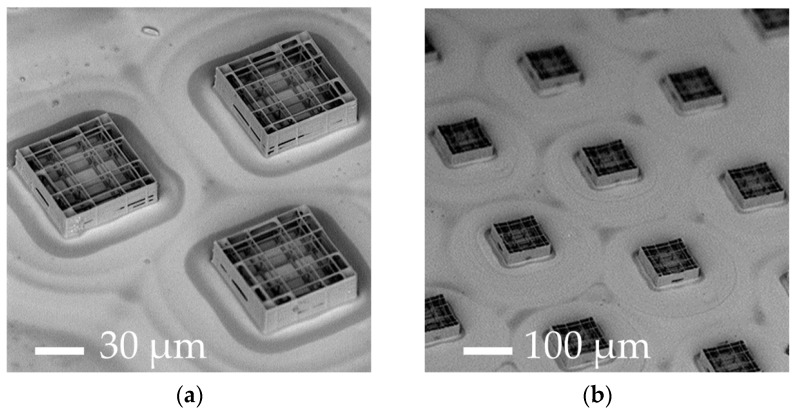

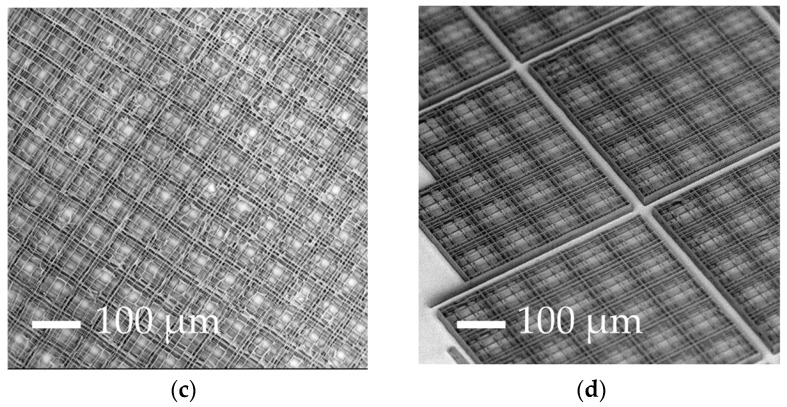
SEM images of the micro-fabrication after the optimization. (**a**) The culture substrate composed of three elementary nichoids patterned in a 200-µm side triangle; (**b**) The culture substrate of elementary nichoids in a hexagonal layout (300 µm side); (**c**) The culture substrate covered with continuously-packed nichoids (CPN), which share external walls with the adjacent ones; (**d**) The culture substrate covered with a fractionated supermatrix of nichoids (FSN), including 218 matrixes at a distance of 30 µm.

As shown in Table 2, for a high average laser power, the fabricated structures were visibly damaged, with interrupted or warped polymeric lines (DAMAGED in Table 2). Conversely, for high scan speeds, the amount of energy per unit of time delivered to the material was too low so that 2PP did not occur (Ø in Table 2). Some of the tested writing parameters led to unstable microstructures which partially or completely collapsed (UNSTABLE in Table 2, Figure 2), while others resulted in stable nichoids (STABLE in Table 2, Figure 3), thus enabling us to identify several useful combinations of fabrication parameters. However the reproducibility in the fabrication of the structures decreased with the increasing scan speed when fabricating a large number of structures for a long time. In addition, it is worth noting that 2PP is a threshold nonlinear phenomenon. Indeed, the radical polymerization in the resist may occur only if the irradiated energy per focal volume is above a certain threshold and below a damage threshold. This explains why both laser average power and scan speed influence the damage/non-polymerization of the photosensitive material. Examples of non-optimized fabrication outcomes are shown in Figure 2.

Referring to the initial configurations with few elementary nichoids (Figure 1d,e), the main drawbacks were the long micromachining time and the detachment and misalignment of grids which led to a structural collapse of the nichoids and, thus, an inability to accurately control the 3-D microgeometry (Figure 2a,b).

A mathematical model was developed [54] to predict the dependence of the transversal diameter *d* and the height *h* of the polymerized voxel on the average laser power *P* and the exposure time *t*:

(1) *d* = *K* × [ln(*P*^2^ × *t* × *E*_th_^−1^)]^1/2^

(2) *h* = *K* × [(*P*^2^ × *t* × *E*_th_^−1^)]

where *E*_th_ is the threshold energy for the 2PP process and *K* is a constant that depends on the properties of the material and the experimental setup. However, this model requires the empirical determination of the parameter *K*, and its predictive ability lies within a narrow and specific processing window (i.e., above the 2PP threshold energy and below the damage threshold) [55]. In this work, we used a trial and error procedure, which was systematic, simple, and enabled us to quickly obtain reliable results.

### 3.2. Increasing the Percentage of Glass Surface Covered by the Nichoids

Using the optimized parameters, we were able to structurally fabricate stable nichoids with a reduced machining time. The first configurations with elementary nichoids arranged in a triangular layout (relative distance 200 μm) (Figure 1d) and in hexagonal distribution (300 µm side) (Figure 1e) resulted in a good architectural stability and an optimal control of the 3-D geometry of the nichoids (Figure 3a,b respectively).

Having reduced the elementary nichoid manufacturing time to 30 s, we were able to fabricate almost 400 nichoids onto the whole glass slide, covering approximately 10% of the cell culture surface in 3 h (Table 1, column B). Considering a typical occupation of about 20 cells/nichoid [49], we could theoretically obtain around 8000 nichoid-cultured cells per sample in this configuration (Table 1, column B).

**Figure 4:**
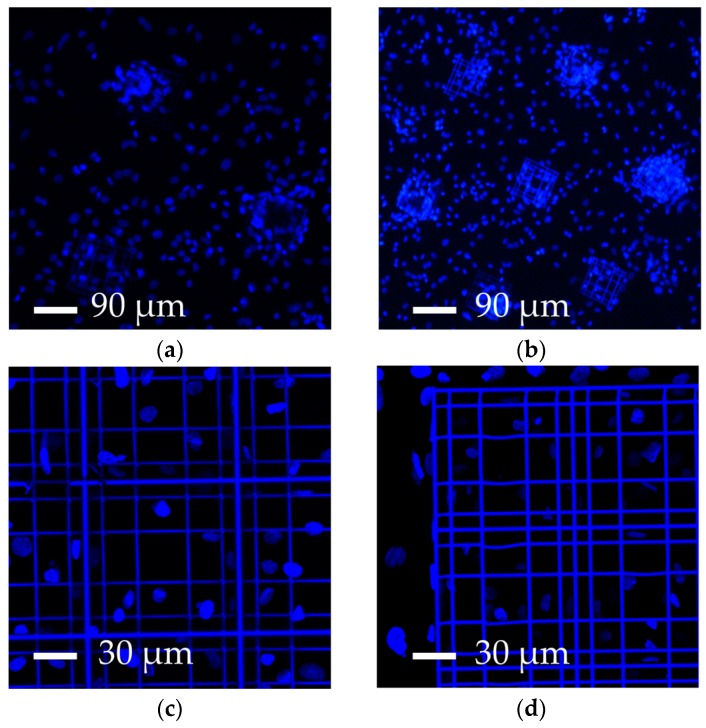
Fluorescence images of cell-populated niche substrates. (**a**) Elementary nichoids patterned in a 200-µm side triangle; (**b**) The culture substrate of elementary nichoids in a hexagonal layout (300 µm side); (**c**,**d**) The culture substrate covered with a fractionated supermatrix of nichoids (FSN), including 218 matrixes at a distance of 30 µm. Nuclei are stained in DAPI (blue).

Despite such technological improvements, the percentage surface coverage of the 3-D structures was still too low, so that biological measurements were inevitably diluted by the cells deposited on the flat glass surface surrounding the elementary nichoids, which did not experience the 3-D nichoid environment. Thus, in order to decrease the 2-D unpatterned surface between the nichoids, we designed a continuous scaffold CPN (Figure 1f and Figure 3c) by fabricating longer and continuous lines instead of fabricating the nichoids one by one. This new design reduced the manufacturing time for an elementary nichoid to 18 s. Therefore, by using the following parameters 12 mW–1 mm∙s^−1^, we fabricated almost 3500 adjacent nichoids in a machining time of 17 h, covering 100% of the treated culture surface (Table 1, column C). However, the CPN structure (Figure 1f) also showed structural instabilities (Figure 2c). In fact, even a single defect inside the structure has negative effects on the stability of the whole scaffold because of its packed configuration. Moreover, a manufacturing time of 17 h was incompatible with a potential industrial exploitation, and it was difficult to remove the unexposed photoresist from the internal lattice of the nichoids as the solvent can only be accessed from the top. Therefore, although this configuration produced very nice samples (Figure 3c) which could theoretically provide 70,000 nichoid-cultured cells (Table 1, column C), these drawbacks caused us to abandon the CPN design. To overcome the above issues, we developed the FSN configuration with 168 matrixes, each composed of 5 × 5 nichoids, spaced by 80 µm [56]. With this layout, the residual tensions due to the volumetric shrinkage and local defects in the microstructures did not affect the whole 3-D architecture. This was due to the 80 µm spacing that made each matrix a stand-alone entity, not subject to the problems of the neighboring matrixes. Initially, the FSN structure was fabricated in 12 h of machining time with the following parameters 12 mW, 1.5 mm∙s^−1^ [56]. Then, by further increasing the fabrication speed to 3 mm·s^−1^, with a laser power of 13 mW, we were able to reduce the fabrication time for an elementary nichoid to 7 s (Table 1, column D). This was possible because, by tracing longer continuous lines, the translation stages have more time to accelerate, thus reaching a higher velocity. In order to increase the number of nichoids, we reduced the distance between the matrixes of nichoids to 30 µm. Thus, thanks to these technological improvements, we were able to fabricate culture substrates by including more nichoid matrixes (i.e., 218) (Figure 1g) than the previous 168 matrixes in the first FSN configuration [56] with the same machining time of 12 h (Table 1, column D). The results were also very good in terms of structural stability and precise control on the 3-D geometry of the 2PP structures (Figure 3d). We were able to extend the laser-treated surface of cell culture from a circle with a 3-mm radius to one with a 4-mm radius. Thus, this last FSN configuration enabled us to cover up to the 88% of the surface of cell culture with the nichoids and to obtain an estimated number of 10.90 × 10^4^ cells experiencing the nichoid environment (Table 1, column D). The larger number of nichoid-cultured cells will enable us to achieve a more homogeneous cell population and thus obtain a more reliable sample for further quantitative analyses including the investigation of gene expression. However, this latter configuration also initially had some instability issues due to focus variations onto the non-perfectly planar glass surface. We observed differences in the *Z*-position of the glass surface ranging between 6 µm to 10 µm from the center to the edges of the substrate, which were not negligible with respect to the structure height (30 µm) and thus caused the nichoids to detach from the glass samples or collapse on themselves (Figure 2d). We pre-characterized the sample surface and modified our control software by implementing a point-by-point compensation of the focus in the vertical direction to balance out the glass concavity. In addition, we increased the structure height and initiated the irradiation deeper inside the glass by 8 µm, to ensure that the manufactured blocks anchored on the substrate surface. All these actions enabled us to achieve stable structures (Figure 3d). The optimal processing window was rather narrow and was sensitive to uncontrolled variations in the laser characteristics (pulse duration, wavelength, and spot size) and in the photosensitive resin (different batches, ageing). Therefore, after the initial optimization, we had to periodically re-optimize the processing window by finding optimal values that changed slightly but always came close to the ones here presented. Finally, to confirm the cell adhesion on all the nichoid culture substrates, human bone marrow-derived MSCs were seeded and stained with DAPI (Figure 4a–d) [49,50].

### 3.3. Comparison with Results from the Literature

Cells in our engineered nichoids can adhere to a 3-D environment, experiencing a high surface-to-volume ratio (Figure 4a–d), can be easily extracted/detached by standard culture protocols (e.g., trypsin detachment), and can be imaged both live and fixed by (inverted) optical and confocal microscopes. In fact, the nichoids were laser written directly on standard glass coverslips (150 µm-thick). There are studies in the literature dealing with 3-D culture systems for SC expansion, including gelatin [28], collagen-I [29], fibronectin [30], Polyethylene-glycol (PEG) [31], Poli-L-Glycolic Acid [32,33], hyaluronic acid [34], vitronectin [35], poly(N-isopropylacrylamide)-PEG [36,37], and carboxymethyl-hexanoyl chitosan [38] in the form of hydrogels, nanofiber scaffolds, and other 3-D structures [21,26]. However, since cells were typically embedded in packed cross-linked matrices, imaging them by optical microscopy or collecting viable cells either for further analysis or for potential therapeutic applications would be difficult and, in some cases, almost impossible. In addition, our nichoid-based culture substrate is made of a chemically stable and biocompatible photoresist, so that cell-material surface interactions can be neglected.

The evidence of multipotency maintenance without any chemical supplements reported in [52] was attributed to the interaction between cells and the 3-D nichoid structure. This is an interesting issue from an industrial and clinical perspective, because of the minimization of cell manipulation and associated risks for the host. This is a great advantage over the most recent literature in which chemical exogenous factors were used for this purpose [8,9]. Finally, the FSN culture substrate was demonstrated to preserve the pluripotency of mouse ESCs without either soluble factors (i.e., LIF) or a feeder layer [56]. These results are in agreement with other studies in which 3-D polymer-based scaffold systems for stem cell culture made up of biodegradable matrices have been used [3,20,33], but with the advantages previously outlined. A recent work proposed a fabrication method that produces poly (ε-caprolactone) (PCL) nanofibers over a large culture surface, by pressing the PCL substrate against a femtosecond laser fabricated glass mold [41]. The authors reported pluripotency maintenance and cell detachment by trypsin. However, the low geometrical control of the microstructured surface did not allow a fine study of the effects of the mechanical stimuli exerted on the cells. Indeed, cells grow along the bent nanofibers, instead of being inside a regular 3-D scaffold matrix.

### 3.4. Future Prospectives

#### 3.4.1. Further Reduction in the Machining Time

2PP is intrinsically a serial micro-fabrication technique. In order to massively increase the production of samples for industrial applications, a simple optimization of laser manufacturing parameters would not be effective because of the speed and acceleration limitations of the current high-precision translation stages. We observed that at higher scan speeds, the machining time was not proportionally reduced due to the significant fraction of time occupied by repositioning the focus between writing steps, which is already performed at the highest speed. A solution to improve the production rate might consist in a soft lithography technique to replicate microscopic structures, namely micro transfer molding (µTM) [57]. However, the internal network of the structures would be irreversibly damaged when extracted from the mold. There are studies in the literature dealing with the resolution of this problem, such as membrane-assisted micro-transfer molding (MA-µTM) [58], but the high complexity of 3-D geometry of the nichoids limits the use of this technique.

To further decrease the micro-machining time and increase the culture surface covered by nichoids, an attractive solution consists in parallel processing, in which multiple foci are created from a single laser beam, using optical systems such as a microarray of lenses [59], a holographic spatial light modulator, and digital micro-mirrors [60]. With these systems, the multiple spots created would be focused into the same objective, allowing the polymerization of multiple lines simultaneously, and decreasing the fabrication time by a factor equal to the number of spots. All the spots would be localized within the field of view of the objective, which in our case is larger than one nichoid, and translating the sample, lines of any length could be polymerized. Another interesting method to decrease the fabrication time could be to integrate a galvanometer scanning mirror system which uses moving magnets for fast and precise positioning of mirrors for the deflection of laser beams, leading to a rapid and accurate scanning of the spot in the focal plane. Closed-loop galvanometer systems have a frequency response up to 10 kHz, and can provide a constant velocity of beam deflection and fast-step response times in the 100 µs range [61]. This could bypass the problem of the limited acceleration of the stages and thus reduce the machining time, while maintaining the same high resolution of our current 2PP apparatus. Galvanometer mirrors are routinely used for beam scanning in the focal plane in high speed confocal microscopy, in combination with high numerical aperture objectives. The drawback of this approach would be the limited field of view of our high numerical aperture objective that would compel us to stitch different parts together to fabricate structures of several hundreds of micrometers, and the impossibility of galvanometric scanners to trace vertical lines (along the *Z*-axis) unless coupled with mechanical stages or piezoelectric systems for the vertical displacement. Indeed, galvanometric scanners allow the focus position only in the *X*-*Y* plane.

#### 3.4.2. Decreasing Cell Adhesion in the Nichoid Surroundings

So far, we have neglected the fact that the surface of the glass substrate is not completely treated by the laser writing process. In fact, as shown in Figure 1f,g, an annular untreated region is left at the edge of the substrate. This region is not structured to enable the sample to be fixed to the holder during the irradiation process, and afterwards to enable sample manipulation with tweezers without damaging the scaffolds. To avoid cell adhesion to this surface, before culturing the cells, we applied a PDMS ring with the same shape. This operation is however rather complicated as the ring is positioned manually with the risk of ruining the edges of the scaffolds. In the future, we plan to reduce the fraction of the cells not experiencing the 3-D nichoid environment using substrate glasses with a hydrophobic coating. These substrates will prevent cell adhesion not only on the external annular region, but on the whole flat substrate, thus favoring homing and 3-D suspension of the cells in the nichoid microstructures.

## 4. Conclusions

In this work, we have reviewed our activity on the upscaling of 2PP-fabricated 3-D substrates for the expansion of stem cells, by increasing the surface coverage and reducing the machining time. We have discussed how, by suitably tailoring the irradiation parameters, we could reduce the fabrication time of a single nichoid from 30 min to 7 s, achieving the most recent result of 88% of the surface of cell culture covered by nichoids. This result will enable the majority of the stem cells to grow in the 3-D scaffold. We thus expect to obtain more evident biological data to confirm our previous hypothesis that a truly 3-D culture substrate, allowing 3-D isotropic cell adhesion, can drive stem cell responses to allow cell multi/pluripotency without supplementing any chemical media and/or feeder. We plan to further improve our culture system by increasing the surface patterned with nichoids by a parallelization of the manufacturing process, and by removing the 2-D areas by depositing a non-fouling (e.g., hydrophobic) coating.

If successful, this system could be used for the expansion of patient-specific stem cells for clinical treatments, such as customized cell therapies for degenerative diseases such as Alzheimer’s and Parkinson’s diseases.

## Figures and Tables

**Table 1:** Technical parameters regarding the various nichoid layouts and upscaling processes. A = three elementary nichoids, B = 367 elementary nichoids, C = continuously-packed nichoids (CPN), D = fractionated supermatrix of nichoids (FSN).

Parameters	A	B	C	D
Total machining time	1.5 h	3 h	17 h	12 h
Power–scan speed (mW–mm∙s^−1^)	15 ^1^–0.01	12 ^2^–1.5	12 ^2^–1	13 ^2^–3
Elementary nichoid writing time	30 min	30 s	18 s	7 s
Surface of cell culture (mm^2^)	0.24	28.27	28.27	50.26
% of surface covered by the nichoids	10%	10%	100%	88%
Number of nichoids	3	367	3500	5450
Estimated nichoid-cultured cells/sample	60	8000	7 × 10^4^	10.9 × 10^4^

^1^ Ti:Sapphire laser. ^2^ Yb:KYW laser.

**Table 2:** Optimization of the scan speed (mm·s^−1^) and laser power (mW) resulting in the process window for the microfabrication of niches. STABLE = structurally stable niches; UNSTABLE = structurally unstable niches; DAMAGED = structurally damaged niches; Ø = no polymerization occurred.

Scan Speed (mm∙s^−1^)	Power (mW)	12	13	14	15
**1**		STABLE	DAMAGED	DAMAGED	DAMAGED
**2**		STABLE	DAMAGED	DAMAGED	DAMAGED
**3**		UNSTABLE	STABLE	STABLE	DAMAGED
**4**		UNSTABLE	STABLE	STABLE	STABLE
**5**		UNSTABLE	STABLE	STABLE	STABLE
**6**		UNSTABLE	UNSTABLE	STABLE	STABLE
**7**		Ø	UNSTABLE	STABLE	STABLE
**8**		Ø	UNSTABLE	UNSTABLE	UNSTABLE
**9**		Ø	Ø	UNSTABLE	UNSTABLE
**10**		Ø	Ø	UNSTABLE	UNSTABLE
